# Update and inclusion of the Institutional Animal Care and Use Committee approval in the Asian-Australasian Journal of Animal Sciences

**DOI:** 10.5713/ajas.2019.0001ED

**Published:** 2019-08-23

**Authors:** Cheol-Heui Yun

**Affiliations:** 1 Department of Agricultural Biotechnology, College of Agriculture and Life Sciences, Seoul National University, Seoul, 08826 Korea; 2 Deputy Editor-in-Chief, Asian-Australasian Journal of Animal Sciences, Seoul 08776, Korea

Institutional Animal Care and Use Committee (IACUC) in conjunction with animal welfare issues are becoming critically important in applying regulations and laws about research project using animals. The research projects at any institution where the animals are used for federal grant as well as for most, if not all, private sector funds, mandate the proper act and use of IACUC.

Recently, there has been a tremendous attention and public awareness from outside of the research community on the necessity of animal experiments. Therefore, research scientists working with animals have been asked for various moral engagement of their work to make sure to obey the proper use of animals for continued scientific contribution in the areas that critical to societies and general public [1].

Accordance with public awareness and demanding, the AJAS is planning to update the checklist by adding the IACUC approval item for the manuscript submission as following;

Institutional Animal Care and Use Committee (IACUC) approval: All animal experiments should be reviewed by the Institutional Animal Care and Use Committee (IACUC) or similar committees of the organization at which the experiment was carried out.

*Please provide approval number*: _________________________

It is hope that those action will improve the way authors proceed with animal researches for the care and use of animals based on the 3Rs (Replacement, Reduction, and Refinement, which are often important from a legal, ethical and scientific standpoint), and to well-acquainted with animal welfare issues (i.e., ethically and scientifically justified and that animals are treated responsibly and humanely).
